# Damage‐associated molecular patterns and coagulation

**DOI:** 10.1111/bjh.70258

**Published:** 2025-11-16

**Authors:** Jun Yong, Cheng‐Hock Toh

**Affiliations:** ^1^ Department of Clinical Infection, Microbiology and Immunology University of Liverpool Liverpool UK; ^2^ The Roald Dahl Haemostasis and Thrombosis Centre Liverpool University Hospitals NHS Foundation Trust Liverpool UK

**Keywords:** coagulation, damage‐associated molecular patterns, histones, innate immunity, neutrophil extracellular traps, thrombin

## Abstract

Damage‐associated molecular patterns (DAMPs), released into the extracellular space following tissue injury, are increasingly recognised as potent procoagulant molecules integral to haemostasis and the pathogenesis of thrombosis. Their procoagulant influence spans all phases of the cell‐based model of coagulation while simultaneously extending beyond traditional haemostatic pathways through direct modulation of inflammatory and innate immune responses. By coupling coagulation and immunity, DAMPs drive the self‐perpetuating cycle of immunothrombosis characteristic of many critical illnesses. Targeting this underexplored interface offers the promise of novel diagnostic and therapeutic approaches, particularly in conditions where coagulopathy coexists with hyperinflammatory states.

## Graphical abstract

Injured cells release damage‐associated molecular patterns (DAMPs), which promote clot formation by activating coagulation, inflammation and immune pathways. Targeting these immunothrombotic mechanisms, via DAMPs, offers new opportunities for diagnosis and therapy in thromboinflammatory disease.

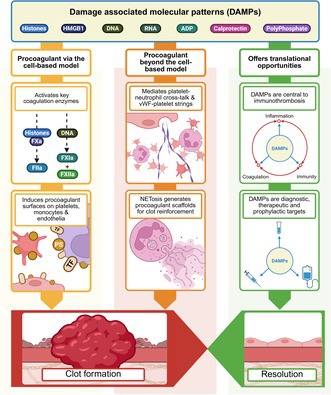

In a nutshell, damage‐associated molecular patterns (DAMPs) are clinically relevant procoagulant molecules that play pivotal roles in haemostasis and thrombosis.

## WHAT ARE DAMPS?

DAMPs are a heterogenous group of molecules that have vital intracellular functions but become potent responders to injury when released extracellularly. DAMPs elicit a broad spectrum of host responses aimed at preserving vascular integrity and containing microbial invasion.

Well‐characterised DAMPs include:

1. *Nuclear constituents*, such as DNA, histones and high mobility group box‐1 (HMGB1), which are among the most extensively studied.^1^, ^2^
2. *Extranuclear molecules*, such as RNA, adenosine diphosphate (ADP), polyphosphates (linear polymers of inorganic phosphate) and calprotectin, a cytoplasmic regulator of myeloid function.^2^

DAMPs were originally characterised as mediators of inflammation and innate immunity but are now recognised for their pleiotropic procoagulant effects.^3^ They modulate the kinetics of coagulation enzymes and the bioavailability of procoagulant surfaces, notably activated platelets and novel interfaces such as neutrophil extracellular traps (NETs). These extruded web‐like structures, comprising an amalgamation of nuclear and cytoplasmic content, serve as potent biophysical and biochemical accelerators of coagulation, further linking immune activation and coagulation.^4^

## HOW ARE DAMPS PROCOAGULANT?

The cell‐based model (CBM) of coagulation delineates three overlapping phases—initiation, amplification and propagation, all of which are influenced by DAMPs^2^:

### DAMPs initiate coagulation

The CBM emphasises tissue factor (TF) bearing surfaces as the primary igniter of coagulation.

- Extracellular histones increase endothelial permeability by disrupting intercellular junctions or inducing cell death, thereby exposing subendothelial TF.
- HMGB1 and histones induce TF expression in endothelial cells and monocytes. Histones decrypt TF by promoting phosphatidylserine externalisation and protein disulphide isomerase expression.
- Circulating histones can substitute for Factor (F)Va as a co‐factor in the prothrombinase complex in a reaction that is phospholipid independent.

### DAMPs amplify coagulation

The initial TF‐triggered thrombin spark is insufficient for clot formation and requires amplification by the platelet–von Willebrand Factor (VWF) axis.

- HMGB1 and histones mobilise VWF from endothelial Weibel–Palade bodies.
- HMGB1, histones, ADP and calprotectin directly activate platelets, which release additional DAMPs (e.g. ADP, HMGB1 and polyphosphates) to create a self‐amplifying thromboinflammatory loop.
- Polyphosphates, DNA and RNA activate FXII and FXI, thereby recruiting the intrinsic pathway.

### DAMPs propagate coagulation

Negatively charged phospholipids (e.g. phosphatidylserine) provide procoagulant surfaces that enable the assembly of tenase and prothrombinase complexes to accelerate thrombin generation towards clot formation.

- Extracellular histones induce phosphatidylserine expression in endothelia, monocytes and erythrocytes.
- Extracellular histones generate TF and phosphatidylserine‐enriched microparticles, which act as mobile procoagulant platforms.
- Integration of histones and DNA into fibrin fibres enhances clot strength and confers fibrinolytic resistance.

## HOW DO DAMPS EXTEND THE CELL‐BASED MODEL OF COAGULATION?

### DAMPs interfere with endogenous anti‐coagulants

- HMGB1 and histones bind thrombomodulin (TM) to interrupt protein C (PC) activation.
- Histones bind to glycosaminoglycans, including heparan sulphate, to reduce anti‐thrombin activity.
- Histones autoactivate FVII‐activating protease (FSAP), which degrades TF pathway inhibitor (TFPI).

### DAMPs interconnect coagulation with immunity

- Histones and HMGB1 trigger NET formation to create procoagulant platforms that are enriched in DAMPs and neutrophil proteases, which degrades TFPI.^5^, ^6^ Integration of NETs within the clot architecture confers biomechanical strength and fibrinolytic resistance.
- HMGB1, histones and calprotectin mediate bidirectional cross‐talk between platelets and innate immune cells.^7^ Platelet‐derived HMGB1 stimulates NETosis and monocytic TF expression. Reciprocally, neutrophil‐derived calprotectin and histones enhance platelet thrombogenicity.
- DAMPs promote VWF–platelet string formation and downregulate VWF‐cleavage through ADAMTS‐13 degradation by thrombin and neutrophil elastase. VWF‐platelet strings further potentiate clot formation through tethered leucocytes and erythrocytes.^2^, ^6^
- DAMPs are indirectly thrombogenic through proinflammatory interleukin release, which upregulates circulating fibrinogen levels (interleukin [IL]‐6), induces TF expression on monocytes (IL‐1β and tumor necrosis factor [TNF]‐α), activates platelets (IL‐8 and ‐12) and suppresses TM and the endothelial protein C receptor (EPCR).^2^

### DAMPs and its integral role within coagulation

- Thrombin, activated protein C (APC), FSAP and plasmin proteolytically degrade histones. Thrombin can also cleave HMGB1.
- Fibrinogen and TM directly bind and neutralise histones. TM and PC attenuate HMGB1 signalling through the receptor for advanced glycated end products (RAGE).
- In tissue repair, histone 2B‐protease‐activated receptor‐1 (PAR1) interactions promote plasmin‐induced cell migration. Through RAGE, HMGB1 promotes the migration and proliferation of keratocytes, fibroblasts and endothelia.^1^, ^2^

## HOW ARE DAMPs CLINICALLY RELEVANT IN HAEMOSTASIS AND THROMBOSIS?

### DAMPs in immunothrombotic disease

- DAMPs are central to the pathophysiology of immunothrombosis.
- Their clinical significance was highlighted during the coronavirus disease 2019 (COVID‐19) pandemic, where severe disease was characterised by co‐existing hyperinflammation and hypercoagulation.^8^
- The immunothrombotic paradigm predates COVID‐19 and is recognised as a common mechanism underlying poor outcomes in patients on the intensive care unit with sepsis, trauma and acute pancreatitis.^5^, ^9^, ^10^
- In chronic inflammation, such as in inflammatory bowel disease and connective tissue disease, excessive DAMP release promotes immunothrombotic complications.^11^, ^12^

### Translational potential of DAMPs in coagulation

- Immunothrombosis forms the basis of many therapeutic conundrums in coagulation medicine whereby conventional approaches fail to recognise the complex multidirectional cross‐talk between coagulation, inflammation and innate immune activation.
- This underpins the inadequacies of conventional anti‐platelets and anti‐coagulant therapeutics, which are constrained by bleeding risk and fail to adequately address the upstream drivers of immunothrombogenesis.
- A salient example is the lack of benefit from escalated dose heparin therapy in mitigating thrombotic risk among critically ill COVID‐19 patients.

### DAMPs and clinical management opportunities

- The shortcomings of traditional anti‐coagulant strategies underscore the need for novel management approaches that selectively modulate DAMP‐driven pathways. Their potential utility as diagnostic, prognostic and therapeutic biomarkers is being investigated in both acute (e.g. sepsis, trauma) and chronic (e.g. lupus, cancer) conditions.^13^, ^14^
- Multiple anti‐DAMP strategies are in preclinical development, including non–anti‐coagulant heparins that neutralise histone‐mediated cytotoxicity in sepsis. Harnessing DAMPs as biomarkers may also enable therapeutic repurposing of innate anti‐coagulants with anti‐DAMP properties (e.g. TM and APC).^3^, ^13^
- Alternatively, extracorporeal DAMP removal is already being used clinically, although current results are equivocal at best and warrants further evaluation.^15^

## CONCLUSION

This article highlights how DAMPs are procoagulant and positions the DAMP–immunothrombosis axis as a promising and underexplored frontier in coagulation medicine. Targeting these molecular sentinels may offer a transformative approach to mitigating thrombotic and inflammatory complications across a spectrum of critical and chronic illnesses.

## AUTHOR CONTRIBUTIONS

J. Yong and C. H. Toh contributed equally to the conceptualisation of this review. J. Yong reviewed the literature base and drafted the manuscript and figures. J. Yong and C. H. Toh contributed equally in revising and finalising the manuscript and figures for submission.

## CONFLICT OF INTEREST STATEMENT

J. Yong and C. H. Toh declare no competing financial interests.

## Data Availability

The data that support the findings of this study are available from the corresponding author upon reasonable request.
